# Environmental Factors Influencing Help-seeking Behavior Among Japanese Adolescents

**DOI:** 10.14789/jmj.JMJ22-0046-OA

**Published:** 2023-11-29

**Authors:** EMI IIZUKA

**Affiliations:** 1 University of Yamanashi Faculty of Medicine, Yamanashi, Japan; 1 University of Yamanashi Faculty of Medicine, Yamanashi, Japan

**Keywords:** help-seeking, adolescent, mental health, original questionnaire survey

## Abstract

**Objectives:**

Previous studies have demonstrated that adolescents do not tend to actively engage in help-seeking behaviors. Therefore, it is imperative to create an environment where adolescents can seek assistance on their own. However, no concrete method to create such environments has been established.

**Design:**

We studied adolescents’ help-seeking behaviors by administering a questionnaire that collected information on who offer help (“helpers”), how help is offered (“methods of help”), and where these interactions occur (“places of help”).

**Methods:**

We asked college students to recall their thoughts related to seeking help when they were 10-15 years old.

**Results:**

Our results indicated that adolescents require trustworthy helpers who respect and understand them, face-to-face interactions, peer helpers of a similar age, mental health dialog, and safe and secure location outside of school for seeking help.

**Conclusions:**

This study suggested a method to provide assistance in the field of child mental health, which is crucial for the development of the adolescents' ability to seek help and resolve mental health problems on their own.

## Introduction

Adolescents do not tend to actively engage in help-seeking behaviors^1)^. A study on middle and high school students reported that 38% of students did not seek help from others even when they were troubled^2)^. Another study on junior high school students revealed that >50% of students did not seek assistance in cases of trouble; moreover, it indicated that help-seeking behaviors varied depending on the type of concern, with >90% of students seeking assistance for some concerns^3)^. A 2019 survey^4)^ conducted by the Japan Cabinet Office reported that >40% of participants aged 15-29 years answered “somewhat disagree” or “disagree” to the question “Is there someone in your family you can talk to about any problems?”

Numerous studies have identified factors that discourage help-seeking behaviors. For instance, a literature review identified four factors: (1) demographic factors, such as sex, age, education, and income; (2) network variables, such as social support; (3) personality variables, such as self-esteem and self-disclosure; and (4) severity of the problem and symptoms caused by the problem^5)^. Another study identified (1) sex-based differences, (2) availability and number of helpers, (3) mental health status, and (4) professional involvement^6)^ as factors that influenced adolescents’ help-seeking behaviors.

Other studies have examined the decision-making process of seeking help^7-9)^. To seek help, an individual must pass through various stages, including “awareness of the problem,” “recognition of the need for assistance from others,” and “decision- making for requesting assistance.” Instead of identifying psychological factors that prevent individuals from seeking help, some studies focused on educating individuals on how to request help, including how to communicate extreme distress (i.e., “SOS”)^10)^ and gain literacy education^11, 12)^. All these approaches focused on adolescents.

Some studies have identified helper-specific (i.e., those who help) factors that affect help-seeking behaviors of individuals. Helpers should have the skills to address adolescents’ wide spectrum of needs by offering new or expanded services and collaborating with other service providers if necessary. Researchers have discovered that many adolescents lack trustworthy and reliable confidants even when they are willing to interact with an adult close to them. These responses reflected a shortage of comfortable help-seeking environments for adolescents. Other factors such as the availability of physical facilities and equipment were also identified as influencing factors. Researchers have recommended training the staff to tend to various needs of the youth, offer new or expanded services, and establish appropriate collaborations with other service providers^13)^. Many adolescents felt that attitudes of the staff affected their access to social support. Other adolescents reported feeling unwelcomed in such spaces^13)^. Furthermore, a lack of staff who can empathize with adolescents, are sensitive to their needs and realities, and know how to listen and talk to them hinders the usage of existing healthcare services by adolescents^14)^.

These studies suggest that solely approaching adolescents and offering help do not necessarily create a desire to seek help. Therefore, it is crucial to create comfortable environments for adolescents to ask for help. Although the need for such spaces has been identified, specific measures necessary for creating such spaces have not been established. To counteract this issue, three environmental factors must be considered: helpers, methods of helping, and places of helping. Each factor must meet the specific conditions desired by adolescents, and these conditions should be urgently identified.

This study aimed to clarify the conditions required to encourage adolescents to seek help. To accomplish this, we administered a questionnaire to college students that focused on helpers’ attitudes, methods of helping (e.g., remote or face-to-face), and places where an adolescent is more likely to seek help. College students enrolled in this study were asked to recall their experiences when they were 10-15 years old and describe their current conception regarding this.

## Materials and Methods

### Participants

The principal investigator conducted this study in June 2021. The study participants were second- and third-year university students enrolled in the B and C Faculties of the University of A. No exclusion criteria were applied. All participants were explained the purpose, method, and ethical implication of this study, and receiving a duly filled questionnaire from participants was considered their consent to participate in the study.

### Questionnaire

The questionnaire used in this study enquired the views of the participants at the time of the survey by asking them to recall their feelings regarding help-seeking behavior when they were 10-15 years old. The questionnaire comprised questions about the following items: 1. gender, 2. experience of seeking help, 3. potential helpers, 4. ideal helpers, 5. ideal counseling place, 6. counseling methods (face-to-face and online), 7. peer support, and 8. support they wish they had received. Responses were given in a yes-or-no response format and free-text answers. For the yes-or-no type questions, respondents were asked to provide reasons for their answers in the form of open-ended questions. Since this was a self-administered questionnaire, a preliminary survey was conducted to ensure that respondents understood the questions as intended in the questionnaire design. Multiple open-ended items were added to gain a more specific understanding of what adolescents sought for when they wanted help.

There were three main reasons for selecting university students as the research subjects: they can objectively verbalize their feelings, they are considered to have relatively vivid memories of the time when they were 10-15 years old, and they can decide whether to participate in the research of their own volition.

The survey responses were recorded using a combination of written and Google forms.

### Statistical analysis

The responses to the selected items and reasons for the responses were tabulated, and responses to the open-ended questions were classified and categorized by question item per the KJ method^15)^. The specific procedures used in the KJ method are described below. (1) Label creation: For the data obtained from free descriptions, a response with one sentence was entered on a card as one item. For responses with multiple sentences, the sentences were separated to ensure the intended meaning was retained, and each sentence was entered on a single card. (2) Cards that were qualitatively similar were grouped together. Each group was given a name that could be expressed in simple words. (3) Group formation (large category): The groups created in (2) were further grouped together with other related groups to form a large group. Each group was given a new name that succinctly expressed the image of the group. In this process, all inappropriate responses were excluded.

### Ethical considerations

The purpose and content of the study were verbally explained to the participants, and they were informed that participation in the study was voluntary and that questionnaire responses were anonymized. Questionnaire completion was regarded as consent for study participation. This study was conducted after obtaining approval from the Research Ethics Committee of the Juntendo University Graduate School of Health and Sports Sciences (2021-14).

## Results

### Participant

This study included 211 university students (58 men and 153 women).

### Helper characteristics

The labels were grouped into 7 major categories: “a person who listens to me,” “a person who respects me,” “a person I trust,” “a person who offers me affirmation,” “a person who helps me sort out my feelings,” “a person who understands me,” and “a person who can keep a secret” (Table 1).

**Table 1:** Conditions of the person you wish to consult (help-giver)

Broad category	Medium category	Labels (Typical/excerpt)
A person with listens to me	listen to me	to the end, without interrupting, just (listen), carefully, properly
A person with respects the me	sincere attitude	kind, serious, not mocking
	a predisposition to take the client point of view	being an ally, being there for me, looking out for me, they worry about me
	open-mindedness	not judging, do not impose values
A person with relationship of trust	someone you can trust	we can trust each other, credible, be reliable
	relax one's guard (around)	expose yourself, I can tell you anything
A person with affirm the me	not to deny	not to deny, affirmation
	to accept what I have to say	to accept what I have to say, empathy
A person with help me sort out my feeling	someone to think with me	someone to think with me, they think of me
	objective advice	advice after listening to you, they say all my good and bad points right
A person with who understands me	accepts me	accepts me, they recognize me
	understands me	he understands how i feel. they understand me
A person with can keep secre	be able to maintain a secret	tight-lipped, I will keep it to myself. I do not tell other people

For the category of “a person who listens to me,” the responses were “till the end,” “without interrupting,” “just listens,” “carefully,” and “properly,” which indicated the individual’s willingness to listen to the client.

For the category of “a person who respects me,” available responses reflected a sincere attitude toward the client (e.g., “kind,” “serious,” and “not mocking”), predisposition to consider the client’s viewpoint (e.g., “being an ally” or “being there for me”), and tolerance (e.g., “not judging” and “looking out for me”).

For the category of “a person I trust,” we noted expressions such as “someone you can trust” or “relax one’s guard (around).” These responses indicate the potential for building a trustworthy relationship.

For the category of “a person who offers me affirmation,” the desired attitude was “not to deny” and “to accept what I have to say.” In other words, this indicated the acceptance of the client’s perspective.

For the category of “a person who helps me sort out my feelings,” the respondents desired for “someone to think with me” and “provide objective advice,” considering their position as a third party consultant.

For the category of “a person who understands me,” the respondents expressed a desire for someone who “accepts,” “understands,” or “grasps” the request for help. The respondents also desired someone who would try to understand them, rather than just focus on what they discussed.

### Characteristics of places of help

The respondents classified places where they could seek help into two broad categories: “assurance of safety” (e.g., assurances of confidentiality and no “leaks”) and “assurance of security” (e.g., environment for one-on-one consultations where individuals can calmly confide in each other) (Table 2).

**Table 2:** Conditions of the place you want to consult

Broad category	Medium category	Labels (Typical/excerpt)
Assurance of safety	Other people will not know that you are consulting	be able to maintain a secret, no information leakage, content is not leaked,
	The content of the consultation will not be leaked	no one else can hear you, nobody is here, no one will see it, no one else will know, one by one
Assurance of Security	One-on-one consultation	one-to-one, peace of mind
	Calmly confide in each other	everyone can enter without discrimination, quiet and relaxing

Upon being asked “do you think it would be good if there were places outside of school where students could feel free to seek advice or have a place to stay after school?,” 204 respondents (96.7%) answered “yes,” whereas 7 (3.3%) answered “no.” Most respondents confirmed (responded “yes”) the existence of a place outside of school where they could seek advice. The respondents who answered “yes” were asked to further classify their responses into four major categories: “Having a place to stay makes me feel safe,” “Nonschool locations are better locations for talking,” ”More people and opportunities to talk,” and “Expanded relationships and sense of values.”

Most respondents believed that university students should volunteer in schools to build relationships with students, with 169 (80.1%) responding “yes.” The reasons for responding “yes” included “Close in age and easy to discuss,” “Easy to talk to as a third party,” “Important to be able to discuss,” “Can talk about own experiences,” and “Was meaningful to me.” We did not obtain sufficient negative responses for further analysis.

### Characteristics of methods of help

**Ease of consultation:** Upon being asked “Which is easier: seeking help using a smartphone or tablet (i.e., internet or remote consultation) or through face-to-face communication?,” 82 (38.9%) participants responded “internet consultation” and 120 (56.9%) responded “face-to-face communication.” Thus, direct communication was preferred over indirect communication as a method of consultation.

**Expectations for resolution:** Upon being asked “Do you think it is easier to solve a problem through online or face-to-face consulting?,” 22 respondents (10.4%) answered “online,” whereas 172 (81.5%) answered “face-to-face.” One major category was obtained for why a respondent believed “internet consultation” was more likely to solve a problem: (It is) easy to say.

Other respondents believed that solving problems “face-to-face” was easier because this method made it “easier to convey feelings and facial expressions,” “easier to feel safe and talk to others,” and “easier to read others’ facial expressions.” Thus, they preferred face-to-face communication.

**Desired supports:** The following open-ended question was asked: “What kind of support would you have liked to have received?” The answers to this question were categorized under the broad category of “an environment which made it easier to ask for help.” Looking at the middle categories and labels, the results for “attitude of the helper,” such as “listening,” were similar to those for “characteristics of the helper,” as described above. In the “mental care” category, “cognitive-behavioral therapy-type involvement” was preferred, such as “changing the way they think” and “how to deal with problems.” The respondents also desired “meeting with everyone,” “one-on-one,” and “regular meetings,” among other forms of involvement that valued each individual (Table 3).

**Table 3:** Desired support

Broad category	Medium category	Labels (Typical/excerpt)
An environment where you want to consult	Prepare the attitude of the person you are helping	listen to me, sincere attitude
	Peer supporter	listening to experiences, consulting with older adults close to your age, adult opinions, broad perspective
	Value each individual	noticing changes, talking to them, regular interviews, one-on-one meetings,
	Mental health care	dealing with stress, knowing how to change your thinking, correcting cognitive distortions, accepting emotions, accepting hard times, and recovering from them, consultation with specialists, cognitive-behavioral therapy involvement

## Discussion

This study aimed to clarify factors related to helpers, methods of helping, and places of help to promote help-seeking behavior among adolescents. The original questionnaire used in this study included questions about helpers’ attitudes, preferred mode of seeking help (remote or face-to-face setting), and the type of needed help. This study included college students who were asked to recall their own help-seeking experiences when they were 10-15 years old and describe their current thoughts regarding the same.

Previous studies^16-18)^ have revealed that “Confidentiality is guaranteed by helpers,” “I am treated with respect by helpers,” “I talk to helpers I trust,” “I talk to helpers who have had the same experience,” “I have helpers who listen to me when I ask for help,” “I have helpers who accept me,” and “I have helpers who respect me” promoted help-seeking behaviors in adolescents. This finding is generally consistent with the current study findings regarding the desired characteristics of helpers. These findings suggest that these seven factors also promote help-seeking behaviors in Japanese adolescents, thus strengthening the validity of the research methods used in this study.

The current and previous studies^19)^ have indicated that “trust” plays a particularly important role in the helper-help recipient relationship. “Trust” in this context implies that when the recipient requests assistance, the helper will understand the problem they are facing, provide useful assistance, and maintain the confidentiality of the consultation. This finding is also consistent with the seven aforementioned factors. Furthermore, the responses to the open-ended questions provided insight into what these participants wanted from helpers, and it was found that they did not want helpers to solve their problems but rather to have positive acceptance of them and understand to their feelings of pain, anger, and anxiety.

Objective advice was also assessed. This did not include preachy imposition of adult values, such as “this is the way to do it.” Instead, it included helping recipients seeking advice from people who accepted them and experienced similar feelings themselves. Ideally, helpers shared their experiences and accurately conveyed the points that were good or should be changed to ensure the best possible outcome. Thus, a mechanism is needed that encourages helpers and help recipients to think together.

Most respondents preferred face-to-face interactions and believed that these were more likely to provide actual solutions as it is “easy to convey feelings and facial expressions,” “reassuring and easy to talk to,” and “easy to read the other person’s facial expression.” Despite these advantages, many respondents reported that remote or internet-based consultations would be “easy.”

Our results demonstrated the effectiveness of peer support received from university students (support from close helpers who have similar problems or have experienced similar problems in the past) was high due to the following factors: “easy to talk to because of their close age,” “easy to talk to as a third party,” “important to be able to ask for help,” and “able to talk about their own experiences.”

When we asked what kind of support respondents wished they had received, they mentioned the following factors: “mental health care,” “creating an environment in which it was easy to ask for help,” “regular meetings,” “one-on-one meetings,” and “improving the attitude of those providing assistance.”

Before the survey began, we predicted that most respondents would prefer remote or phone- or internet-based consultations, because these technologies are commonly used by the current youth population; however, the actual responses indicated that face-to-face consultations were preferred by them. Most respondents also believed that “face-to-face meetings were more likely to provide solutions.” Therefore, many young individuals feel that their problems can only be truly understood in a face-to-face setting. In other studies^20, 21)^, young individuals expressed awareness of the risks of online help, including privacy concerns. Additionally, some individuals expressed concern over the impersonal nature of telecommunication tools, and some believed that the help they received in such settings is unreliable or untrustworthy. This concern may have contributed to our results regarding the places of help.

Furthermore, “regular meetings” and “one-on- one meetings” were desired by respondents in our study; however, prior research^22)^ has shown that the presence of a caring adult is important. Thus, we believe that it is necessary to watch over each individual, regardless of whether they have problems. Care should be taken to monitor their growth and efforts and recognize and respect them.

Moreover, we observed a desire for “mental health care” among youth. Importantly, this was not a request for knowledge regarding mental health but for dialog using methods such as cognitive-behavioral therapy to correct cognitive distortions (which have a therapeutic aspect) and stress management. The respondents expected that these activities will lead to self-reflection, changed perspectives, and psychoemotional growth with a broader perspective and healthier mindset. Social skills training, assertion training, and stress management are forms of mental health care that have been introduced in school settings and reported to be effective^23)^. However, rather than teaching “the correct way” to do something, helpers should incorporate these methods into their counseling and dialog with young individuals. This approach allows young individuals to directly experience the benefits of help-seeking.

Regarding places of help, most respondents sought “assurance of safety” and “assurance of security.” Approximately all respondents (96.7%) requested a nonschool location for the following reasons: “having a place to stay makes me feel safe,” “it is easier to talk outside of school,” “more people and opportunities to talk to,” “more people to talk to,” and “more human relationships and values.” Maximizing the abovementioned factors may help promote help-seeking behaviors. Prior research^24)^ has indicated lack of trust to be a major barrier to seeking help, in addition to concerns about confidentiality and trust^25)^, which may be related to stigma. The fear of broken confidentiality is rooted in the fear of stigma and embarrassment when peers or family members discover that a young person has sought help for a mental health problem. Thus, we believe that creating “consultation places outside of school where safety and security are guaranteed” is necessary for adolescents. Importantly, such spaces should be staffed by trusted helpers.

This study suggested a method to provide assistance in the field of child mental health, which is crucial for the development of the adolescents’ ability to express SOS and resolve mental health problems on their own.

### Limitations of the study

In this study, college students were asked to reflect on their experiences at ages 10-15 years; thus; the study results are subject to recall bias from a convenience sample of college students. Future studies should focus on directly studying adolescents’ responses following the implementation of appropriate processes for parental or caregiver consent.

## Funding

The author received no financial support for the research.

## Author contributions

EI contributed to drafting, planning, data collection, analysis, and manuscript writing.

## Conflicts of interest statement

The author declares that there are no conflicts of interest.

## Availability of data and materials

The data supporting the findings of this study are available from the corresponding author EI upon reasonable request.
